# Temporomandibular trauma and reflections on personal evaluation

**DOI:** 10.1007/s12024-023-00745-9

**Published:** 2023-11-21

**Authors:** Ana Corte-Real, João Abreu, José Pedro Figueiredo, Tiago Nunes

**Affiliations:** 1 Faculty of Medicine, University of Coimbra, Coimbra, Portugal; 2 Clinic and Academic Centre of Coimbra, Coimbra, Portugal; 3 Laboratory of Forensic Dentistry, Faculty of Medicine, University of Coimbra, Av. Bissaya Barreto, Bloco de Celas, 3000-075 Coimbra, Portugal

**Keywords:** Somatic symptoms, Disability evaluation, Trauma, Temporomandibular joint disorders, Adults

## Abstract

The International Consortium Network/Orofacial Pain Special Interest Group focuses on temporomandibular disease diagnosis procedure guidelines as a reference iQAn this scope. Concerning this reference, the aim of this study was to comprehensively analyze injury and sequela frames within European and American approaches to personal damage. A quasiexperimental pilot study of Portuguese orofacial trauma cases in a medico-legal evaluation database was performed with an interrupted time series design. The clinical data were recorded following five criteria of TMJ diagnosis (jaw opening, pain, anatomical deficit, functional deficit, clinical sounds, and occlusal deficit) under three degrees of severity. The injury frame evaluation was recorded in the first-degree stage in all criteria. Pain, as a sequela, was the criterion present in 45% of the sample as spontaneous (20%) or stimulated (25%). Temporomandibular trauma damage evaluation emphasizes the accurate injury diagnosis and sequela framework. Orofacial trauma analysis should focus on the inclusion or exclusion of a TMD diagnosis. This study suggests revising the reference tables on personal damage, considering the inclusion of TMD and its categorization and impact.

## Introduction

Orofacial trauma poses substantial challenges in personal damage assessment [1, 2]. From a collective perspective, it is a public health issue associated with higher health costs [2, 3]. Once the scientific community highlighted its economic impact, this impact was attributed to the failure to meet society’s expectations [1–3]. From an individual perspective, personal damage negatively impacts individual quality of life and well-being [4–6] in terms of anatomical, functional, and psychological damage, as emphasized by the specific anatomical area and physiopathology of injuries and sequelae, which are aspects covered in civil law evaluations [7].

The main etiology of trauma has changed from road accidents to interpersonal violence in the last decade [2]. This might be explained by the socialization and construction of an individual’s personal identity, which is commonly permeated by factors (such as power, aggressiveness, and masculinity) that in turn facilitate engagement in episodes of aggression [4]. Trauma may also be related to falls and iatrogenic procedures [5–7]. Evaluations of disability and impairment caused by orofacial trauma encompass dental or bone damage, including temporomandibular joint (TMJ) diseases [4, 8–11]. Orofacial trauma occurs mainly in men (60.7%) over 40 years old [4, 5]. Comparatively, females show an increase in TMJ trauma, with such injuries not involving dental or facial areas [4, 8], which can be justified by related symptoms that impact the global frame injury context [8].

Temporomandibular disorders (TMDs) correspond to musculoskeletal conditions distinguished by pain and/or dysfunction of the TMJ, masticatory muscles, and associated tissues or structures [12, 13]. According to Ryan’s systematic review, this is the most common orofacial pain condition of nondental origin [14]. Its reported prevalence varies across studies, with peak rates up to 16% and 75% (according to formal diagnostic criteria and self-report surveys, respectively) between the ages of 25 and 45 [14]. Based on guidelines suggested by the International Consortium Network/Orofacial Pain Special Interest Group related to TMDs (DC/TMD), the diagnostic criteria for TMDs consist of two axes: one for physical diagnoses (Axis I) and the other for assessment of psychosocial status and pain-related disability and their respective instruments (Axis II incorporating the Patient Health Questionnaire with 15 topics–PHQ-15 self-report) [12, 14]. According to the DC/TMD group, the clinical evaluation of TMJ is performed by sequential criteria: self-report and clinical status evaluation (jaw opening, lateral and protrusive jaw excursions and sounds, muscular and joint palpation, referred pain, and imaging analysis) [12–14]. The multifactorial etiology of TMD is congruous with the biopsychosocial model of illness [14]. It includes tooth loss, occlusal disorders, parafunctional habits, such as clenching and grinding, emotional stress, articular pathology related to direct trauma events, and poor posture [12–15].

According to European guidelines suggested by the European Confederation of Experts in Personal Injury Assessment and Repair (CEREDOC), the medico-legal evaluation of personal damage is referenced in tables with specific sections for stomatological evaluation [15–17]. According to the damage assessment European table, the sequelae related to TMJ trauma are TMJ affectation, mandibular dysfunction, tooth loss, and opening deficit [15]. Although the decision-making process in the legal context relies on the clinical knowledge and expertise of the health professional [17], the absence of a specific item for TMDs precludes uniformization and accurate evaluation. A similar context is stated in American Medical Association (AMA) guidelines, focusing on the impact of TMJ affectation on chewing, speech, lower deformity and pain [18]. AMA highlights discal displacement and articular degenerative procedure as two medical diagnoses following the previous parameters [18, 19].

Therefore, the present study evaluated medico-legal assessments of the temporomandibular joint as affected by orofacial trauma by comprehensively analyzing injury and sequela frames to provide evidence for scientific discussion of its accurate evaluation.

## Methods

### Sample

A quasiexperimental pilot study of the Laboratory of Forensic Dentistry Laboratory (LFD) database was performed as an interrupted time series design. The LFD belongs to the Faculty of Medicine (University of Coimbra, Portugal) and provides orofacial traumatology evaluations for personal damage analysis under European Medical Law (civil, labor, and criminal). The sample was selected from the LFD database records between 2014 and 2022 according to the following inclusion criteria: age between 18 and 65 years; report of orofacial trauma (via falls, interpersonal violence, road accidents or iatrogenic procedures); and temporomandibular injuries and sequela identification at four sequential times of the trauma assessment and personal damage evaluation. The exclusion criteria were systemic, oncologic, and genetic diseases and previous trauma history.

### Study design

The research team comprised health professionals with forensic and clinical habilitations within 8 years of practice in medico-legal evaluation. They proceeded with the examination after being informed about the study’s objectives. Informed consent was provided according to the Declaration of Helsinki on human subjects and in compliance with the guidelines of the Ethics Committee of the Faculty of Medicine (CE-048/2017).

**Fig. 1:**
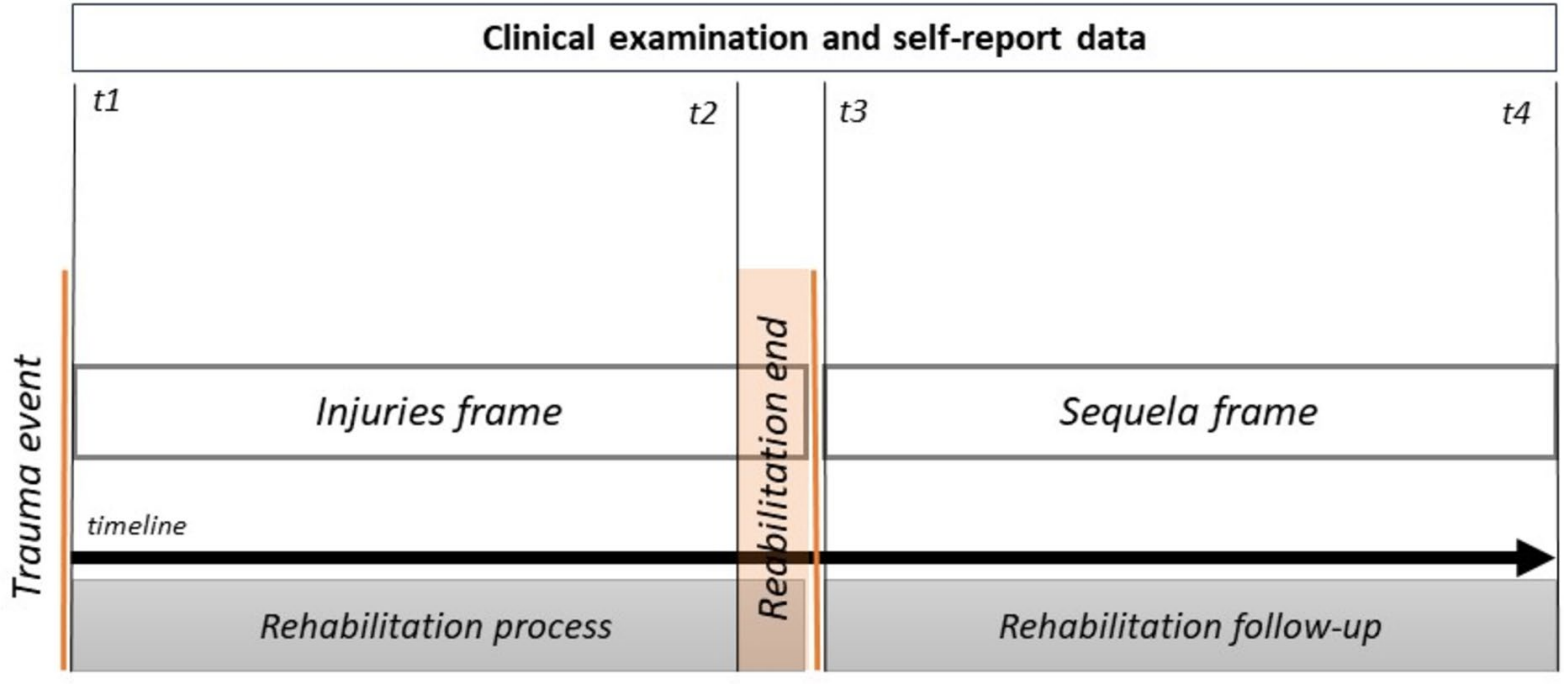
The research methodology

The research methodology (Fig. 1) included TMJ injury frame identification, both after trauma and before the rehabilitation procedure, and TMJ sequela frame identification, after the rehabilitation procedure, into damage management. Consequently, four clinical examinations by the health professional were recorded for each victim in the medico-legal assessment: (t1) after the trauma event for injury frame identification; (t2) injury framework analysis prior to the final rehabilitation; (t3) sequelae frame identification after the final rehabilitation following preliminary medico-legal conclusions; and (t4) follow-up conclusions. Clinical data analysis was performed, recording five criteria (Table 1) regarding TMJ trauma [12, 19–31]: (1) jaw opening under the interincisal opening measure; (2) pain (intangible criterion based on the patient’s valuation), spontaneous or simulated; (3) anatomical deficit or morphological affectation of dental structures, bone and soft tissues by imaging analysis; (4) functional deficit and clinical sound analysis; and (5) occlusal deficit or occlusal interference in dental relation including malocclusion diagnosis according to Angle’s criteria [22].

**Table 1:** Research methodology and data criteria (*concerning the age of the patient (36 for children; 38 for adolescents; 40 for adults)

	**Designations**	**Categories**
**Timeline**	Jaw opening (OI)	I (< 20 mm)
		II (20–35 mm)
		III* (> 36 mm)
	Pain (P)	I (spontaneous) II (stimulated) III (no pain)
		I (dental and bone) II (bone) III (no morphological changes)
	Anatomical deficit (Ad)	
	Functional defict (Fd)	I (two or more functions)
		II (one function)
		III (no function)
	Occlusal defect (Od)	I (bimaxilar)
		II (unimaxilar)
		III (no occlusal changes)

The victims completed a self-report questionnaire (EQ-VAS). The EQ-VAS is a visual analog scale included in the EQ-5D-5 L version (EuroQol Group) [20]. The EQ VAS assesses a patient’s self-rated health. This scale features endpoints marked as “The best health you can imagine” and “The worst health you can imagine.”

A descriptive analysis of the results was performed.

## Results

**Fig. 2:**
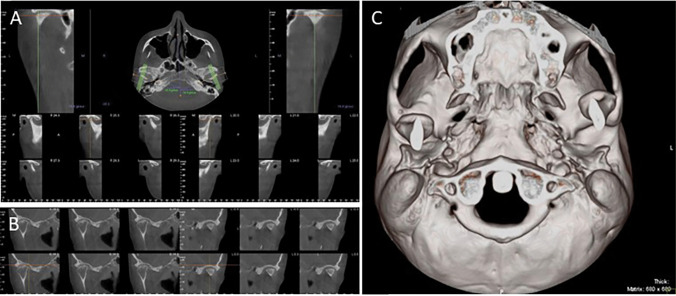
**A** TMD diagnosis documented by DCIM images from CBCT evaluation of a 37-year-old female (sample no. 2). This corresponded to coronal images between horizontal (in the upper) and sagittal slices of the right TMJ (R24.3 to R29.3) and the left TMJ (L20.0 to L25.0) identifying the following anatomic and functional deficits: severe flattening of the condyle and osteophytes, with a reduced joint space and a general increase in density to the glenoid fossa. **B** TMD diagnosis documented by DICOM images from CBCT evaluation of a 57-year-old male (sample no. 45). This corresponded to sagittal images of the right TMJ (R18.5 to 19.6) and left TMJ (L0.0 to 0.8), identifying the following anatomic and functional deficits: severe flattening of the left condyle and osteophytes, reduced joint space, namely, in the right with erosion of the cortex and increased density to the glenoid fossa. **C** TMD diagnosis was documented by tridimensional reconstruction of DICOM images from CBCT of the open mouth of a 42-year-old female (sample no. 66). This corresponds to an abnormal position of the left condyle following a functional deficit in extreme open-mouth activity

The research team selected 70 records and health data (highlighted in Fig. 2). Over 35% (*n* = 20) were reported to have TMJ trauma (Table 2). The mean age of the patients was 41 years (range of 18 to 65 years), and most were female (78%).

**Table 2:**
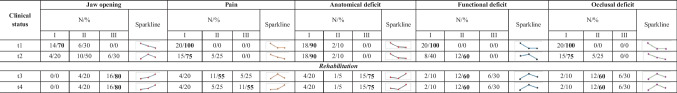
Data analysis following five criteria and four clinical status evaluations (*n* = 20)

Following the early stages, t1 and t2, and after the rehabilitation process, t3 and t4, the jaw-opening valuation corresponded to a sequential increase in the distance between the upper and lower incisors (O’s value). Concerning the first stage of trauma (t1), the pain corresponded to the I-stage in all the victims; stages II and III were recorded after the rehabilitation process in half of the sample and corresponded to the victim’s perception of health quality (Table 3).

**Table 3:** Heal Quality perception by the victim and the *Quantum doloris* evaluation (*n* = 20)

	EQ-VAS			*Quantum doloris*		
	0–50	51–75	75–100	1–3	4–5	6–7
	(*N*/%)			(*N*/%)		
Clinical status						
**t1**	0/0	20/100	0/0	0/0	20/100	0/0
**t2**	15/75	5/25	0/0	15/75	5/25	0/0
Rehabilitation						
**t3**	4/20	11/25	5/25			
**t4**	4/20	8/25	9/55			

In the first stage of trauma (t1), bone and dental anatomic changes correspond to stage I in all victims. In the last stage, the anatomic deficit was overcome (75% of the cases). However, 20% of the cases met the stage I criterion (exemplified in Fig. 2A and B).

Regarding the first stage, at least two functions were affected. One function was involved in 60% of the victims in the forward stage t3 (60%) (exemplified in Fig. 2C), although the rehabilitation procedure ended.

In terms of temporary damage, the individual self-reported perception was recorded following *the Quantum doloris* parameter (Table 3).

## Discussion

The medico-legal evaluation of orofacial trauma encompasses clinical examination of the TMJ and individual self-reported studies [6, 5, 10–12, 22, 25]. TMJ diseases are identified by the WHO in the international disease coding system, namely, ICD-11, highlighting TMDs [13]. The TMD evaluation was focused on guidelines suggested by the International DC/TMD Consortium Network and Orofacial Pain Special Interest Group [12], updated in 2021 [25].

Focusing on guidelines suggested by the CEREDOC group [15], updated in 2010, TMDs are not a sequela identified in medico-legal standard evaluation; indeed, AMA guidelines, updated in 2023, also do not include TMDs as a diagnosis of medico-legal evaluation [18]. The present study emphasizes the diagnosis of TMDs in orofacial trauma, its significance as a relevant sequela, and the need for its inclusion in the reference tables. Although different organizations and jurisdictions may have their own guidelines based on cultural and socioeconomic issues, it is advisable to consult standard guidelines. Understanding the accurate methodology of TMD diagnosis is the basis for personal damage assessment, which introduces the evidential data for discussion in this scope. In the present study, the individual self-report, the selection of criteria for the clinical status (pain; jaw opening; anatomical, functional, and occlusal deficit), and their analysis were recorded with respect to injuries or temporary damage, as well as sequelae or permanent damage, in a holistic connection within the reality of medico-legal evaluation.

Giannakopoulo’s study emphasized that none of the TMJ components is exempt from injury in trauma [21], so it should be an anatomical region that is included in the clinical evaluation of the victim and examined by a health professional qualified to assess personal orofacial damage.

The present study design allowed a longitudinal evaluation of clinical status, with four evaluations impacting the rehabilitation process for personal orofacial damage. As a multifactorial disease with a traumatic etiology, a timeline series of health data records, as a set of measurements taken at intervals over time, represented a methodological procedure to identify an absolute correlation of the individual perception and the professional assessment of the clinical status. Corporal damage was evaluated as temporary in two stages of the clinical process, enabling identification and follow-up assessment of the injuries. Corporal damage as a permanent issue or impairment was assessed after the rehabilitation process as a sequela frame diagnosis. The timeline steps for clinical diagnosis after the rehabilitation process were related to the progression of TMJ pathology, following Zhang’s study [22].

In line with the literature [14, 15], the present findings emphasized the female group in TMJ trauma, according to their impact on the global frame injury context, such as increased sensitivity to pain [8].

Concerning temporary assessment in the early stages of TMJ trauma, the inflammatory stage manifests itself primarily by involving the TMJ components, uni- and/or bilaterally, influenced by the energy involved. In line with the injury frame engaging on bone components, the articular surfaces of the fibrocartilage, the articular disc, and the synovial lining of the joint space, both upper and lower [5, 21], and the role of these structures in supporting the dental arches. Psychosocial affectation can act synergistically within the complexity of orofacial trauma concerning the TMJ [5], involving pain as a transversal complaint and its impact on quality of life. The medico-legal assessment of temporary damage includes the victim’s psychosocial impact on the *Quantum doloris* value [16, 17]. TMJ trauma as a complex injury was carefully studied through two clinical consultations, corresponding to t1 and t2; training the victim as a coevaluator of the clinical condition through a self-questionnaire (EQ-VAS) as a quantitative measure of health outcomes provided insight into the patient’s judgment. This longitudinal analysis allowed the evaluation of the reproducibility of pain as an intangible value related to the psychological state and somatization of the victim. Functional issues associated with TMJ trauma (limitation of opening, limitation in excursive movements, deviation of the opening, and malocclusion manifesting later as a crossbite on the side of the fracture and hypereruption of the teeth on the opposite side) play a temporary role in TMJ trauma, highlighting decreased interincisal value with limited mandibular opening. Speech, chewing, and swallowing are affected [5, 18–20]. These diverse functions are affected by injuries to the TMJ, recorded in the early stages.

Concerning permanent assessment, the pain criterion was related to the victim’s performance in TMD assessments following DC/TMD standards, performing the PHQ-15 [12, 15, 25]. In line with Ryan’s study, a shorter questionnaire increased participation rates, and the EQ-VAS was applied for health-perception valuation in the monitoring of TMD [31]. The present study identified decreased interincisal values after rehabilitation ended, resulting in jaw-opening limitations (25 mm, minimum) based on the following TMD values in the DC/TMD standards: lower than 40 mm for adults, 38 mm for adolescents, and 36 mm for children [12, 25]. Its relation to functional issues (excursive movements) and sounds, malocclusion-engagement, and anatomic changes [4] defines the pattern of TMD and, consequently, the degree of personal damage. Furthermore, the engagement of anatomic-morphological changes in the TMJ components, supporting a higher degree of TMD damage, was identified by cone-beam computed tomography or magnetic resonance imaging [25, 26]. Documentary data are a material and legal proof for medico-legal expert reports and a tool for court declarations, in line with Honey et al. and Corte-Real et al. [28]. Data identifying degenerative TMJ components (namely, the reduction of the interline and rectification of the cortical and osteophytes) can reveal the persistence of anatomic deficits. The findings of anatomical changes, either dental or bone-related or both (5% or 20%, respectively), showed the impact on facial harmony that resulted in a disharmony status. The anatomical changes between the right and left sides, leading to facial asymmetry, impact the medico-legal evaluation of aesthetic damage [17, 19]. Such is emphasized when the trauma occurs in the early stages of individual development, i.e., under the age of 8 years, increasing the damage degree. In the rehabilitation process, the dental prosthetic procedure allows the recovery of dental loss and occlusal function, and it justifies overcoming the dental anatomy deficit. Nevertheless, the occlusal deficit recorded in all the victims was not overcome in 40% of cases, and this could correspond to prosthetic and rehabilitation failure [26]. The occlusal deficit should be evaluated in the TMD diagnosis as either a synergic factor exacerbating the symptoms or a nonrelated feature in TMD pathogenesis [20].

TMJ trauma is extraordinarily complex and impacts the victim’s life, following the global trend to consider TMD as a chronic disease [5, 6, 12, 25, 29–31]. As stated in Gençosmanoğlu’s study, an inappropriate anatomical position in which body structures or segments increase the TMJ load and energy consumption may cause TMD [31]. The originally proposed segmentation of the clinical criteria into three degrees (I to II) allowed future studies on TMD damage categorization and its correspondence with the impairment value of corporal damage. This musculoskeletal disorder engages the individual in a biosocial-psychological context that interacts with risk factors, including depressive and parafunctional disorders, creating a perfect storm in younger people aged 10 to 49 years [2, 12]. In addition, health quality scales, such as EQ-VAS, coengage the victim’s impact as a sequela affecting his or her health quality. Future studies on this topic are needed to present evidence data to the scientific community.

The mandibular dysfunction designation reported in the European reference table corresponds in the reference table to an extensive value of permanent damage, ranging from 6 to 30 points (corresponding to the lower open mouth value, mandibular dysfunction). The expert needs concrete guidance for standardized performance. There needs to be more consistency in the expert assessment, jeopardizing the role of references in the application and orientation of a reference table. TMJ trauma, namely, TMDs, should not be ignored in the clinical analysis of the medico-legal evaluation of orofacial trauma; such trauma must be the object of a critical, reasonable, and experienced medico-legal evaluation. The reference guidelines should consider TMD diagnosis based on the objective criteria presented in this study and should be revised to account for real corporal damage in the medico-legal evaluation of an orofacial trauma scenario.

## Conclusion

In conclusion, this study presents Portuguese data on TMJ trauma assessment, categorizing injuries and sequelae into five criteria for a longitudinal medico-legal evaluation. Greater attention to these data by experts in medico-legal issues is emphasized, especially when considering the absence of TMD diagnoses in the European and American reference tables. The community of professionals should engage in a necessary update of the guidelines to ensure accurate and consistent documentation and reporting of personal damage assessment evidence data.

## Data Availability

Data used to support the findings presented in this study are available on request from the corresponding author.
